# Case Report: Dynamic overlap of melanoma, sarcoidosis, and targeted therapy for *BRAF*-mutant melanoma

**DOI:** 10.3389/fonc.2023.1217179

**Published:** 2023-08-29

**Authors:** Nakul Dar, Sarah E. Gradecki, Elizabeth M. Gaughan

**Affiliations:** ^1^ University of Virginia School of Medicine, Charlottesville, VA, United States; ^2^ Department of Pathology, University of Virginia, Charlottesville, VA, United States; ^3^ Division of Hematology and Medical Oncology, Department of Medicine, University of Virginia, Charlottesville, VA, United States

**Keywords:** sarcoid, sarcoid-like, metastatic melanoma, BRAF inhibitor, MEK inhibitor, targeted therapy

## Abstract

Targeted therapies, including BRAF and MEK inhibitors, are valuable treatment options for patients with unresectable or metastatic BRAF V600-mutant melanoma. With the improvement in survival seen with modern melanoma therapeutics, clinicians are learning the variable patterns associated with extended clinical courses. Sarcoidosis is characterized by non-caseating granulomatous inflammation of unknown etiology, often presenting with cutaneous, lung, or lymph node involvement. There is a known association between sarcoidosis and melanoma, and sarcoidosis is increasingly seen and described in the setting of anti-melanoma therapy. The challenge for clinicians is to differentiate between sarcoid-related and malignancy-related findings, which may follow a variable course over years. We present two cases of BRAF and MEK inhibitor-related sarcoidosis in patients with melanoma and review the literature. The dynamic nature of the clinical and radiographic findings impacted patient management and clinical decisions for years of their treatment course.

## Introduction

The development of effective systemic therapy for melanoma over the last decade has led to marked clinical improvements for patients. While immunotherapy remains the backbone treatment for most requiring medical therapy, the use of combination BRAF and MEK inhibition also results in improved survival for patients with advanced melanoma treated in the adjuvant and unresectable/metastatic settings (1, 2). The optimal selection and sequencing of immunotherapy and targeted therapy in *BRAF*-mutant melanoma depends on patient and disease characteristics.

The main side effects of immunotherapy, termed immune-related adverse events, are the result of dysregulation of self-reactive immune cells. A variety of autoimmune conditions can result, such as dermatitis, colitis, pneumonitis, and endocrinopathy, and management is typically through immunosuppression with glucocorticoids. The primary toxicities of the combination of BRAF and MEK inhibition include drug fever, chills, arthralgia, and diarrhea, which are typically managed by treatment break and dose reduction. Sarcoidosis is a multisystem disease of unknown etiology characterized by the formation of non-caseating granulomas in various organs and has been described both in relation to the presence of melanoma as well as a drug-induced toxicity from anti-melanoma treatments (3–12).

The diverse timing and clinical presentations of sarcoidosis in the setting of malignancy and antineoplastic therapy can be challenging for clinical oncologists. Here, we report two patients who had a dynamic overlap of melanoma and sarcoidosis in the setting of antineoplastic targeted therapy and review the literature. Consent was obtained from both patients prior to inclusion in this report.

## Case 1

We present the case of a 61-year-old woman who was diagnosed with an ulcerated melanoma of the right foot (pT2bN2aM0) in June 2004. Primary treatment consisted of wide excision, complete lymph node dissection following a positive sentinel lymph node, and enrollment in an adjuvant melanoma-peptide vaccine trial. Recurrence in the right iliac lymph nodes was resected in November 2014, followed by adjuvant radiation therapy. The tumor was found to have a *BRAF* V600E mutation. Due to rapid retroperitoneal nodal recurrence, systemic therapy was initiated in March 2015 with high-dose interleukin-2 which failed to prevent disease progression. This was followed by pembrolizumab monotherapy from November 2015 through May 2017 resulting in partial response. Pembrolizumab was held, at patient request, for progressive skin toxicity and escalating constitutional symptoms with disease control through August 2018.

**Figure 1:**
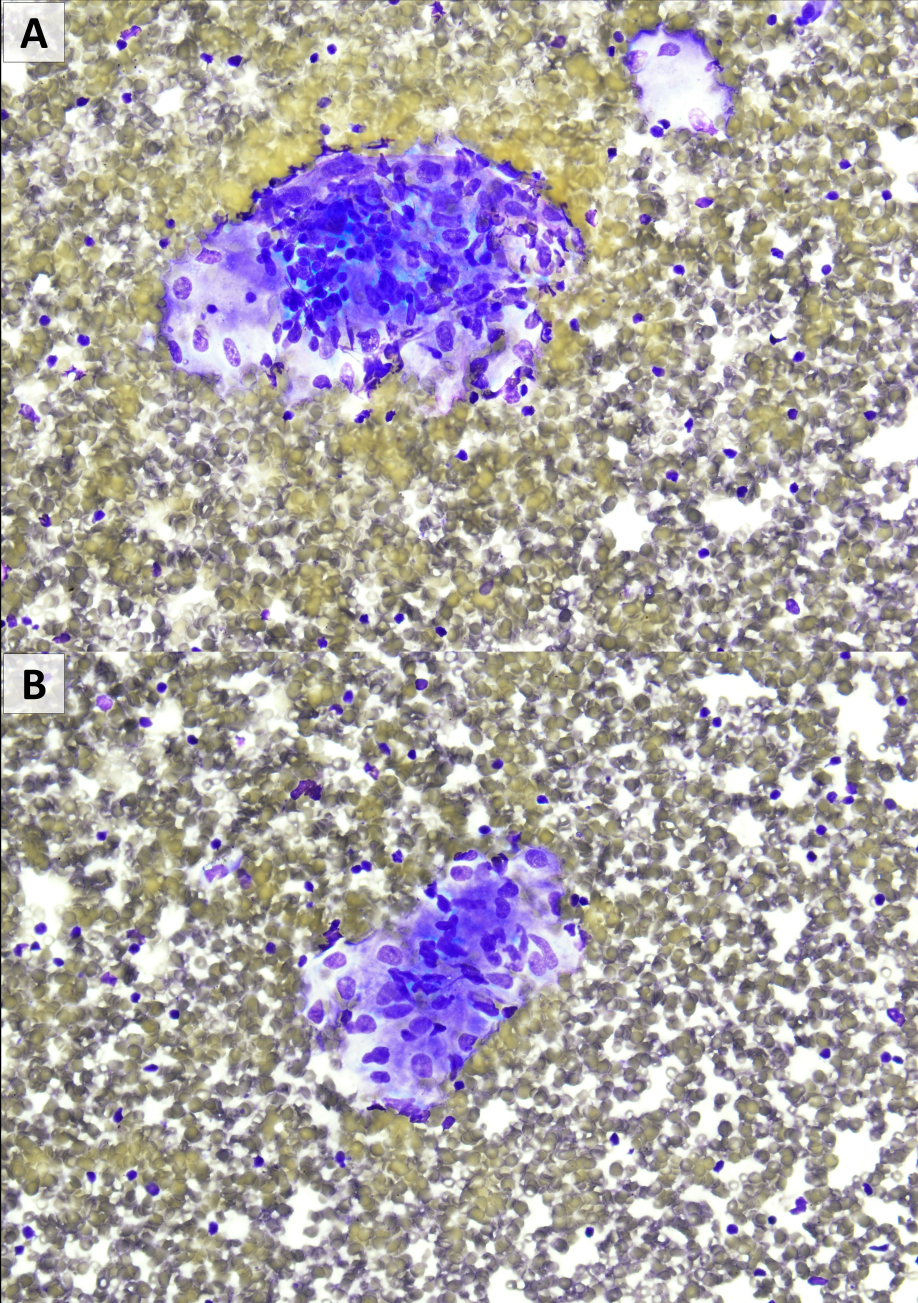
Fine needle aspiration from level 11L showing occasional non-caseating granulomas. There was no evidence of melanoma. **(A, B)**: Diff-Quik stain, 400×)0.

For para-aortic nodal recurrence, the patient started dabrafenib and trametinib in September 2018 and continued through July 2019 with partial response. Treatment was held in July 2019 for marked fatigue, shaking chills, reduced appetite, diffuse pruritus, and development of mediastinal and hilar lymphadenopathy. On examination, she had lower-extremity swelling, erythema of palms, and maculopapular rash. Given radiographic response in known disease and the constitutional symptoms, further diagnostic evaluation of the chest lymphadenopathy was pursued. Endobronchial biopsy of station 11L lymph nodes demonstrated polymorphous lymphocyte population and rare non-necrotizing granulomas (**Figure 1**). Concurrent sampling of station 7 and 4L nodes showed only scant lymph node elements. Discontinuation of dabrafenib and trametinib resulted in rapid resolution of symptoms, and she did not require corticosteroids. The multi-station chest adenopathy resolved without intervention, beyond therapy break, by the follow-up scan at 3 months.

The patient remained off therapy until June 2020 when she recurred in the para-aortic lymph nodes. Treatment with encorafenib and binimetinib was initiated in July 2020 but was limited by toxicity including fatigue, photosensitivity, edema, hypertension, pruritus, and reduced appetite by October 2020 follow-up. Again, her imaging demonstrated response in the known areas of tumor but development of multi-station chest lymphadenopathy. Antineoplastic therapy was held, and her symptoms rapidly improved and lymphadenopathy resolved by a 6-week follow-up scan.

The patient went onto surgical resection of para-aortic disease in April 2021 followed by radiation therapy. Given prior side effects of targeted therapy, the patient opted not to resume systemic treatment. For recurrence, she was restarted on pembrolizumab monotherapy in August 2022. To date, she has tolerated therapy well, without constitutional symptoms. Imaging in July 2023 demonstrated stable disease without evidence of chest lymphadenopathy.

## Case 2

We present the case of a 69-year-old man with *BRAF* V600E-mutant melanoma from an unknown primary who presented with clinically detected right inguinal lymphadenopathy (cT0N1bMx). PET scan revealed an 8-cm right inguinal mass and an incidental 9.5-mm subcarinal lymph node. Endobronchial ultrasound and biopsy of the subcarinal node demonstrated non-necrotizing granulomatous lymphadenitis. The patient had no prior history of sarcoidosis, pulmonary symptoms, skin lesions, or atypical lung infection. His only prior medical history included hypertension and treated localized prostate cancer 4 years prior to the melanoma presentation. The patient started neoadjuvant dabrafenib and trametinib with excellent clinical response after 2 months of therapy, followed by complete superficial right ilioinguinal node dissection. Results from the procedure showed pathologic complete response of the melanoma. The patient then resumed adjuvant dabrafenib plus trametinib combination to complete 1 year of systemic therapy with dose reduction required for drug fever. Imaging after completion of adjuvant therapy had no abnormal findings including resolution of the subcarinal lymph node noted at baseline.

**Figure 2:**
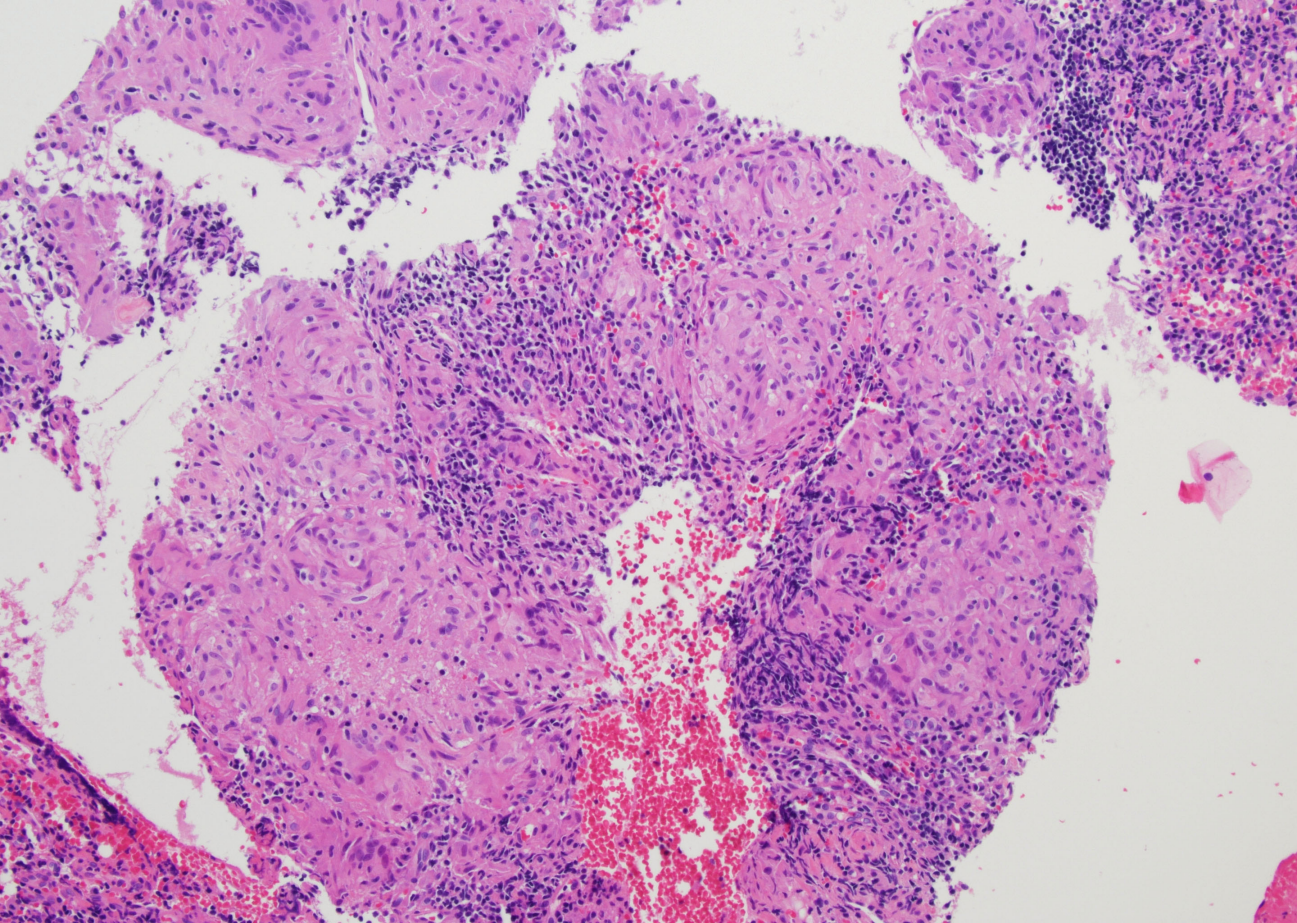
Core biopsy from right inguinal lymph node demonstrating well-formed, non-caseating granulomas. There was no evidence of melanoma. (H&E, 100×).

**Figure 3:**
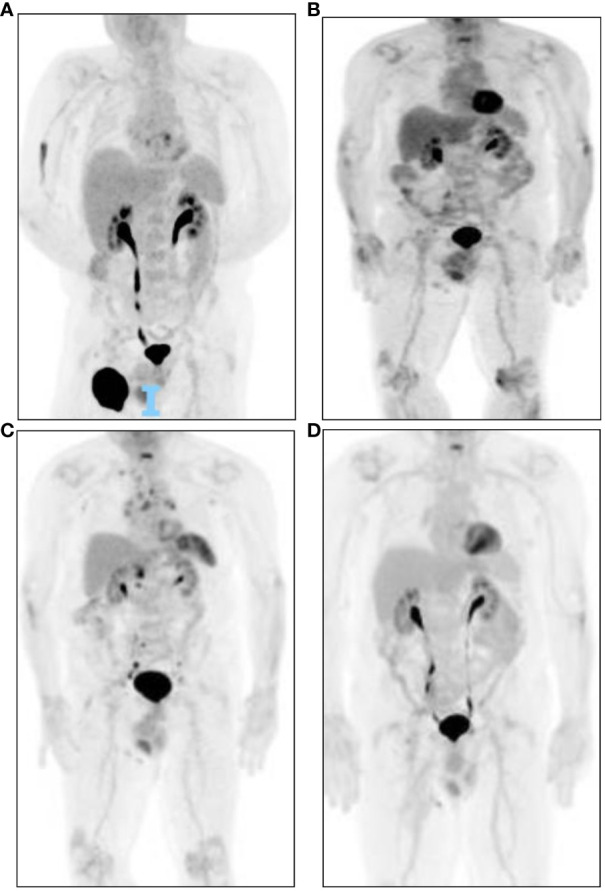
Case 2 PET progression. **(A)** Baseline PET scan on 30/05/2019 showing a large right inguinal mass and prominent subcarinal node with SUV max 2.9. **(B)** PET 28/08/2020, after completion of adjuvant therapy showing postsurgical changes in the right thigh and resolved FDG uptake of the subcarinal lymph node. **(C)** 21/07/2021 representative surveillance PET with mediastinal, abdominal, retroperitoneal, and right iliac lymph nodes (biopsy). **(D)** 28/06/2023, most recent PET. Postsurgical changes without abnormal lymphadenopathy. This patient received no sarcoid-directed therapy over this course.

Surveillance imaging in January 2021 demonstrated multiple new hypermetabolic right supraclavicular, mediastinal, and hilar lymph nodes. There were no concurrent clinical signs or symptoms of melanoma or sarcoidosis. The patient underwent bronchoscopy and endobronchial ultrasound biopsy in January 2021. Pathology revealed non-necrotizing granulomatous inflammation with no evidence of malignancy, and all cultures showed negative results. As the patient was clinically well, he opted for clinical and radiographic surveillance without immunosuppressive therapy. Waxing and waning adenopathy above and below the diaphragm with varying degrees of FDG uptake persisted on imaging. In July 2021, he was noted to have new FDG-avid right inguinal lymphadenopathy. Given proximity to original presentation, biopsy was pursued and demonstrated non-caseating granulomatous inflammation consistent with sarcoidosis (**Figure 2**). The patient, again, opted for clinical and radiographic surveillance. A similar pattern of waxing and waning adenopathy persisted through the last follow-up in June 2023 (**Figure 3**). There has been no recurrence of the melanoma.

## Discussion

Sarcoidosis is a granulomatous disease with variable clinical course that has been associated with some cancers, including melanoma, as well as a side effect of some antineoplastic therapies. Constitutional symptoms, including fever and pronounced fatigue, along with lymphadenopathy, pulmonary nodules, and cutaneous findings are common, with occasional involvement of the liver, spleen, eyes, and heart. Much remains unknown regarding cancer-associated sarcoidosis, but the identification of this clinical entity is important to avoid making cancer-related decisions for a non-malignant etiology. Sarcoidosis is known to spontaneously resolve or remain asymptomatic for many patients, which may account for waxing and waning changes on imaging (13). The interaction of cancer, anti-neoplastic therapy, and sarcoid changes leads to a dynamic clinical presentation that requires awareness throughout a patient’s course.

We, and others, have reported on sarcoid development for patients receiving anti-neoplastic immunotherapy, and clinicians experienced with immunotherapy commonly see reactive lymphadenopathy during patient management (11, 12). There have been reports of sarcoidosis and sarcoid-like reactions to the combination of BRAF and MEK inhibition, described with more frequency in recent years (4, 6–10, 14). Given the less intuitive clinical association between sarcoid-like reactions with these targeted therapies, recognition of this potential differential diagnosis is necessary for contemporary melanoma care. Interestingly, Lheure et al. conducted a retrospective study of 70 patients receiving vemurafenib for a *BRAF*-mutant advanced melanoma, of which five (7.1%) developed sarcoidosis or a sarcoid-like reaction, higher than the normally associated rate for melanoma (15). Huynh et al. conducted a single-center retrospective study of 63 patients receiving dabrafenib and trametinib for BRAF-mutated advanced melanoma and found seven (11.1%) patients diagnosed with sarcoid-like reactions, although none required systemic corticosteroids or treatment discontinuation (7). True incidence rates are challenging to obtain as the clinical presentation and course are highly variable, and it is uncommon to biopsy all radiographic changes.

Non-caseating granulomas are the hallmark pathologic finding in sarcoidosis. These are thought to arise in response to an abnormal immune response to chronic antigenic stimulation (16). The stimulating antigen can vary by person, as can the degree and durability of the inflammatory response. Granulomatous inflammation develops in response to upregulation of activated helper T cells (T_H_1 and T_H_17) leading to the expression of interferon-γ, interleukin-2, and tumor necrosis factor-α (5, 16). The specific pathophysiology between BRAF/MEK inhibitors and development of sarcoid-like reactions is not known. Studies have reported immunomodulatory effects of BRAF inhibitors, including tumor infiltration by CD8+ T cells and increased expression of melanoma antigens (17–20). By influencing antitumor immunity, BRAF inhibitors may have the potential to drive immune-related adverse effects. Further research is needed to better understand the mechanisms underlying a potential relationship between BRAF/MEK inhibition and sarcoidosis.

The clinical course of sarcoidosis is highly variable, and the need for anti-sarcoid therapy is dependent on patient-specific characteristics. Indications for direct sarcoid therapy are related to degree of organ involvement/impairment and systemic symptoms (16). Spontaneous remission is expected for many patients, and, therefore, the finding of sarcoidosis on imaging does not necessitate immediate intervention. Some patients, however, do develop chronic inflammation leading to fibrosis and impact on organ function. For those requiring therapy, glucocorticoids are the first-line approach which can be followed by steroid-sparing secondary agents, as needed. Fortunately, this aligns with general toxicity management guidelines for patients with immune-related adverse events. Adverse events from BRAF and MEK inhibition often improve with a break in therapy, which is a useful potential intervention for patients with limiting symptoms. For cancer patients with treatment-associated sarcoid reactions, oncologists must determine if the findings are sufficient to warrant a change in therapy plan. The identification of a sarcoid-like reaction in an asymptomatic patient does not require interruption of antineoplastic therapy or initiation of immunosuppression. Patients have been able to remain on active antineoplastic therapy, including dabrafenib and trametinib, in the setting of asymptomatic sarcoidosis (7, 8, 20). For our first patient, the systemic symptoms were unmanageable but resolved quickly with holding the BRAF/MEK inhibition.

The patient in our first case received multiple lines of therapy for her metastatic melanoma including both immunotherapy and targeted therapy. There was no history of reactive adenopathy during receipt of her prior lines of immunotherapy, including IL-2 and pembrolizumab. Interestingly, the onset of her sarcoid-like reaction to BRAF/MEK inhibition was nearly 9 months after initiating therapy. Once triggered, she had a clear reactive clinical picture with systemic drug toxicity while receiving BRAF/MEK inhibition with reproducible imaging results with mediastinal/hilar adenopathy, which improved off the targeted therapy. Her disease, manifested as intra-abdominal lymphadenopathy, would reappear as soon as the targeted therapy was discontinued. A high index of suspicion for the varying diagnoses was critical to successful patient management given the different pathologies in the same organ system. We were able to prove the two separate processes through biopsy and then to follow the radiographic and clinical patterns to navigate her treatment. Her symptoms did require break in, and ultimate discontinuation of, her BRAF/MEK inhibition, followed by progressive melanoma. She never required specific treatment for the sarcoidosis. We have, since, been able to reinitiate pembrolizumab with no evidence of the same sarcoid-like reaction.

In our second case, the patient was found to have an FDG-avid subcarinal node with biopsy-proven non-necrotizing granulomas prior to initiation of neoadjuvant dabrafenib and trametinib. The baseline sarcoid of the subcarinal node was asymptomatic and was likely associated with the active melanoma. There were no other points of clinical history that linked to an underlying sarcoidosis diagnosis. He had achieved a melanoma pathologic complete response at surgery and completed his course of adjuvant therapy with resolution of the subcarinal adenopathy. In the 3 years following his adjuvant therapy, his imaging has shown interval development and resolution of lymphadenopathy in the hilum and mediastinum but also in the right inguinal area, the site of his first melanoma presentation. He has undergone frequent imaging studies, and multiple biopsies over this time to track his clinical status rule out recurrent malignancy, which has been an anxiety-provoking and uncertain course. The patient did not have clinical symptoms associated with his radiologic findings, and therefore his case highlights the complexity of a sarcoidosis diagnosis and the need to differentiate between reactive lymphadenopathy and disease recurrence on imaging, including the potential extended timeline and dynamic nature of these issues. His sarcoid findings were always radiographically detected without associated clinical symptoms and spontaneously resolved between scans without steroids.

## Conclusion

We report two cases of patients with *BRAF-*mutant melanoma who were treated with combination BRAF/MEK inhibition and developed a sarcoid-like reaction that complicated cancer management. As evidenced by previous studies, BRAF and MEK inhibitors are immunologically active compounds which, while perhaps contributing to efficacy, may also lead to side effects, it that require close monitoring to distinguish between toxicity and disease progression. Clinicians need to be aware of the dynamic interactions between melanoma, targeted therapy, and sarcoidosis, which can occur any time along the patient course. Given the identification of BRAF V600 mutations in other malignancies, and requisite use of BRAF/MEK inhibition, this clinical picture will likely be seen in the treatment of patients with an array of cancers.

## Data availability statement

The raw data supporting the conclusions of this article will be made available by the authors, without undue reservation.

## Ethics statement

The studies involving humans were approved by University of Virginia Institutional Review Board. The studies were conducted in accordance with the local legislation and institutional requirements. The participants provided their written informed consent to participate in this study. Written informed consent was obtained from the individual(s) for the publication of any potentially identifiable images or data included in this article.

## Author contributions

ND prepared cases and report design, drafted manuscript and edited final version. EG assisted with case identification, report design, manuscript preparation, editing, and revision. SG assisted with pathology figures for the report, and edited and revised manuscript. All authors contributed to the article and approved the submitted version.
